# Ligasure Impact™ to Reduce Complications After Abdominoplasty: A Meta-Analysis of Comparative Studies

**DOI:** 10.1177/15533506251374484

**Published:** 2025-08-27

**Authors:** Salvatore Giordano, Veera Korhonen, Andre’ Salval, Pietro G. di Summa, Carlo Maria Oranges

**Affiliations:** 1 Department of Surgery, 6810 Satasairaala Hospital, Pori, Finland; 2 8058 The University of Turku, Turku, Finland; 3 Department of Plastic, Reconstructive and Aesthetic Surgery, 27230 Geneva University Hospitals, University of Geneva, Geneva, Switzerland

**Keywords:** abdominoplasty, complications, ligasure impact, bipolar electrothermal vessel sealer, body contouring, massive weight loss, duration of surgery, meta-analysis

## Abstract

**Background:**

The optimal dissection technique for flap elevation in abdominoplasty remains debated, particularly in high-risk patients after massive weight loss. The LigaSure Impact™ (LS) vessel-sealing system (Medtronic, Dublin, Ireland) is an advanced energy device used across surgical disciplines to reduce morbidity. This meta-analysis compares LS with conventional techniques to assess its effectiveness in abdominoplasty.

**Method:**

A systematic literature search identified relevant studies comparing LS with standard methods. Primary outcome was the overall postoperative complications’ rate; secondary outcomes included specific wound complications, operative time, and hospital stay.

**Statistics:**

A random-effects model was used for pooled analysis. Risk differences (RD) and 95% confidence intervals (CI) were calculated for categorical outcomes; mean differences (MD) for continuous outcomes. Heterogeneity was assessed using the I^2^ statistic.

**Results:**

The search yielded 3 studies totaling 205 patients. LS significantly reduced overall complication rates compared to controls (RD = −0.46, 95% CI: −0.60 to −0.32, *P* < 0.001). Hematoma and wound dehiscence incidences were significantly lower (*P* = 0.03 and *P* = 0.01, respectively). No significant differences were observed for seroma, infection, or fat/flap necrosis. LS use was also associated with reduced re-operation rates and shorter hospital stays, though operative time was comparable.

**Discussion:**

LS may improve surgical outcomes in post–weight-loss abdominoplasty patients by reducing complications and hospitalization. However, the limited number of studies and small sample size warrant cautious interpretation.

**Conclusion:**

Preliminary evidence suggests that LS may offer potential benefits in abdominoplasty; however, current findings should be interpreted with caution because of limited quality and heterogeneity of available studies. Further research is needed.

## Background

Abdominoplasty is the most frequently performed plastic surgery following significant weight loss, particularly due to the rise in bariatric surgery, with over 1,100,000 procedures worldwide.^1^ This procedure offers benefits such as enhanced quality of life, improved body satisfaction, increased self-esteem, better physiological well-being, and more effective long-term weight management after bariatric surgery.^2-5^

However, compared to other cosmetic surgeries, abdominoplasty has a high complication rate, especially in patients who have experienced substantial weight loss. Common complications include seroma, hematoma, infection, wound dehiscence, healing issues, partial skin necrosis, fat necrosis, damage to the lateral thigh cutaneous nerve, hypertrophic scarring, and systemic issues. The complication rates vary from 4.2% to 50.7%, with re-operation rates reaching up to 43%.^5-17^

Several technical strategies have been proposed to minimize complications and enhance outcomes, such as preserving Scarpa’s fascia, selective flap undermining, combining liposuction, using tissue adhesives, employing internal fixation techniques at closure, and applying postoperative compression dressings. Studies have shown mixed results when comparing techniques for raising the abdominal flap, particularly between scalpel and diathermy methods.^6-20^

In body contouring surgeries, intraoperative tissue dissection is often performed using a monopolar electrosurgical device. While this allows for faster dissection compared to a steel scalpel, it is associated with a higher incidence of surgical complications due to thermal damage to nearby tissues, nerves, and blood vessels. To address this issue and minimize thermal injuries and their related complications, new technologies have been developed. These include energy-based methods that focus on localized effects, sparing the surrounding tissues.

Ligasure Impact (LS, Medtronic, Dublin, Ireland) is an electrosurgical bipolar vessel sealing device that uses mechanical pressure and energy to fuse and cut tissues. It can seal vessels up to 7 mm in diameter and delivers precise amounts of energy during use. Research indicates that LS is safe and effective, reducing intraoperative blood loss and operating time in various surgical fields, including urological, gynecological, colorectal, and general surgeries, when compared to scalpel dissection, clips, conventional diathermy, and other energy-based tools.^21-24^ LS delivers only the necessary amount of energy to the tissues, thereby limiting thermal damage.

However, there is limited research on the use of LS in plastic surgery, and there are only a few prior studies specifically examining its application in abdominoplasty.^25-27^ Furthermore, controversy exists regarding LS’s effectiveness in abdominoplasty to justify its cost.

Thus, we conducted a meta-analysis of comparative studies to address this issue, hypothesizing that LS could offer notable advantages in decreasing not only hematoma rates but also other complications in abdominoplasties.

## Methods

This systematic review and meta-analysis followed the guidelines established by the Preferred Reporting Items for Systematic Reviews and Meta-Analyses (PRISMA) statement, with the corresponding checklist completed.^28^ Each author conducted a comprehensive systematic literature search across multiple databases, including Medline, Cochrane Library, Embase, Scopus, Google Scholar, and ResearchGate, focusing on studies involving the use of LS in abdominoplasty procedures on human subjects.

Search terms included ‘LigaSure’ combined with ‘abdominoplasty’ and ‘body contouring,’ using Boolean operators to refine the search. Additionally, reference lists from relevant articles were reviewed. The search results from each author were consolidated, and duplicate citations were removed.

The search covered the period from inception to August 2025, targeting studies that compared the outcomes of LS use in abdominoplasty procedures against control groups, including both randomized and non-randomized studies. No language restrictions were imposed.

### Search Strategy

We conducted a search and evaluation of studies comparing LS to a control in abdominoplasties. To be included in this review, studies had to fulfill specific criteria based on the PICOS (patients, intervention, comparator, outcomes, and study design) framework. Thus, studies that were non-comparative or involved other types of surgical procedures or outcome measures were excluded. The criteria for inclusion and exclusion are outlined in Table 1. There were no restrictions on ethnicity, patient age, or settings.

**Table 1.** PICOS Criteria for Inclusion and Exclusion of Studies.

Parameter	Inclusion Criteria	Exclusion Criteria
Patients	Patients of any age undergoing abdominoplasty	Patients underwent mini-abdominoplasty or other types of body contouring procedures
Intervention	Use of LigaSure for the dissection in abdominoplasty	
Comparator	Any type of control	Studies comparing different types of instruments
Outcomes	*Primary outcome measure*: hematoma occurrence requiring re-operation. *Secondary outcome measures*: Drainage amount from the wound bed after 24 hours, postoperative seroma, skin necrosis and hypertrophic scarring at follow-up	
Study design	Randomized controlled trials, non-randomized observational trials, retrospective, prospective, or concurrent cohort studies. At least 1 month follow-up	Reviews, expert opinion, comments, letter to editor, case reports, studies on animals, conference reports. Shorter follow-up than 1 month. Studies with no outcomes reported

Two authors (SG and CMO) independently reviewed the abstracts and articles and also examined the reference lists of all relevant studies. To be eligible for this analysis, studies needed to report quantitative outcomes on the use of LS vs control in abdominoplasties.

Each study was independently evaluated by 2 co-authors (SG and CMO) to determine its inclusion or exclusion (see Table 1). Eligible studies had to provide information on baseline characteristics, the type of abdominoplasty performed, and outcomes related to postoperative complications compared to control patients.

### Data Extraction

Data collection was carried out independently by 2 investigators (SG and CMO) and verified by a third investigator (AS) based solely on the retrieved articles, blinded to study authorship. Any discrepancies in the collected data were resolved through consensus among these investigators.

Data were extracted solely from published articles, with no attempt to obtain missing data directly from the authors. However, data from 1 study^25^ were accessible and included in the analysis. The following information was extracted: first author, publication year, study design, number of patients, type of procedure, and primary and secondary outcomes.

The quality of the included studies was independently assessed by 3 investigators (SG, CMO, AS) using the Cochrane Collaboration’s Risk of Bias Assessment tool for Randomized Controlled Trials (RCT),^29^ and the Newcastle-Ottawa Scale for non-randomized studies.^30^ The research team convened to address any disagreements in the assessments and to reach a consensus by discussion.

### Outcomes

The primary outcome assessed was the incidence of any complication as reported in the included studies. Secondary outcomes included specific surgical complications, operative time, and length of hospital stay. The surgical indications and all reported outcomes were documented using the same measurements as provided in the original articles.

### Statistical Analysis

Statistical analyses were conducted using Review Manager 5.4 software (Copenhagen: The Nordic Cochrane Centre, The Cochrane Collaboration, 2020).^31^ Differences in dichotomous outcomes were reported as risk difference (RD), risk ratios (RR) with 95% confidence intervals (CI), while continuous variables were presented as mean differences (MD) with 95% CI. Heterogeneity was evaluated using the I^2^ statistic, which indicates the percentage of total variation across studies attributable to heterogeneity rather than random chance.^32^ Typically, I^2^ values below 25% suggest low heterogeneity, values between 25%-75% indicate moderate heterogeneity, and values above 75% represent high heterogeneity. An I^2^ of less than 75% was considered indicative of non-significant heterogeneity. For the meta-analysis, the Mantel-Haenszel method was applied to dichotomous outcomes, and the Inverse Variance method was used for continuous outcomes.

For dichotomous outcomes such as overall complication rates and hematoma incidence, we calculated both RD and RR, for better clinical interpretation. Where statistically significant risk differences were observed, we derived the Number Needed to Treat (NNT) by taking the inverse of the absolute risk difference (NNT = 1/ARD). This calculation estimates the number of patients who would need to be treated with LigaSure Impact™ instead of conventional dissection methods to prevent 1 additional adverse event. NNT values were rounded to the nearest whole number.

All analyses were conducted using a random-effects model to account for both within- and between-study variability,^32^ given the observational nature of some included studies. Statistical significance was defined as *P* < 0.05. Lastly, sensitivity analyses were performed by systematically excluding each study to assess whether any single study disproportionately impacted the overall results.^33^

## Results

**Figure 1.**
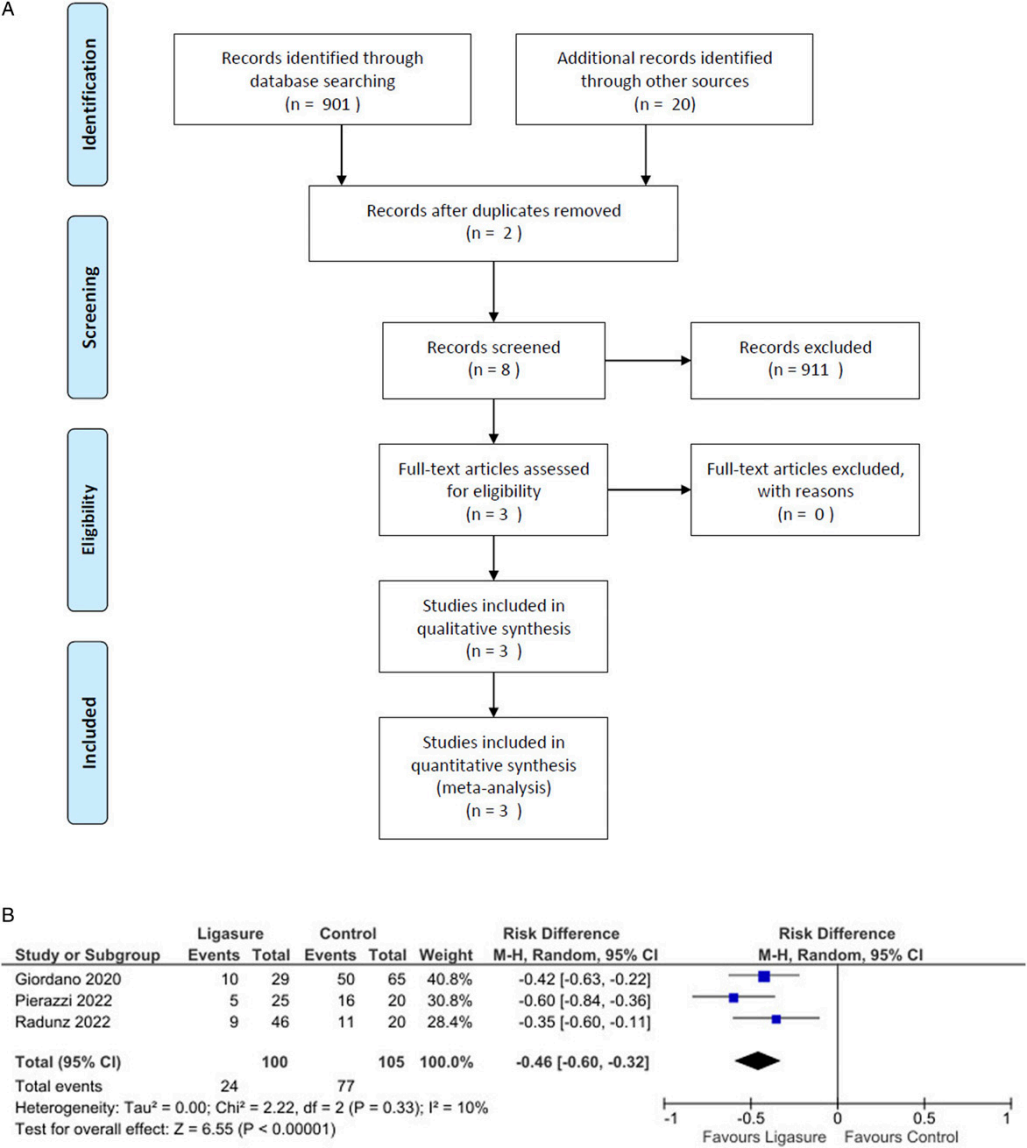
(A): Flow-chart summarizing literature search results; (B): Forest plot showing a significantly decreased occurrence of complications by using Ligasure compared with control.

The literature search identified 901 articles across the previously specified databases, of which three^25-27^ were relevant for analyzing outcomes with LS in abdominoplasty following massive weight loss and in the donor site for microsurgical breast reconstruction (Table 2). The literature search process is illustrated in Figure 1(A).

**Table 2.** Retrieved Studies Included in the Analysis.

Study	Country	Study Type	Years of Study	Group	Sample Size	Age^a^
Giordano et al^26^, 2020	Finland	Retrospective	2008-2015	Control	65	46.6 ± 10.1
				LigaSure	29	43.0 ± 9.9
Radunz et al^27^, 2022	Germany	Retrospective	2011-2012	Control	20	46.5 ± 7.4
				LigaSure	46	46.7 ± 10.2
Pierazzi et al^28^, 2022	Italy	Retrospective	2018-2020	Control	20	45.0 ± 10
				LigaSure	25	50.6 ± 9
				Total	205	

^a^Data expressed as mean ± SD (standard deviation).

The 3 studies,^25-27^ encompassing a total of 205 patients, provided data on postoperative complications. None of the authors appeared to have any financial, commercial, or professional relationship with Medtronic or the manufacturers of LS.

LS was compared only to conventional electrocautery or scalpel dissection techniques, thus, other devices were not mentioned. Postoperative protocols, including antibiotic use, drain management, and wound care, were not standardized across studies.

Pooled analysis revealed a significant reduction in complications in the LS group compared to the control group (Risk Difference = −0.46, 95% CI: −0.60 to −0.32, *P* < 0.001; Figure 1(B); Risk Ratio = 0.37, 95% CI: 0.26 to 0.54, *P* < 0.001, I^2^ = 0%). For overall complications, the absolute risk reduction was 0.53, resulting in a Number Needed to Treat (NNT) of 2. This indicates that treating 2 patients with LS prevents 1 complication compared to traditional dissection methods.

**Figure 2.**
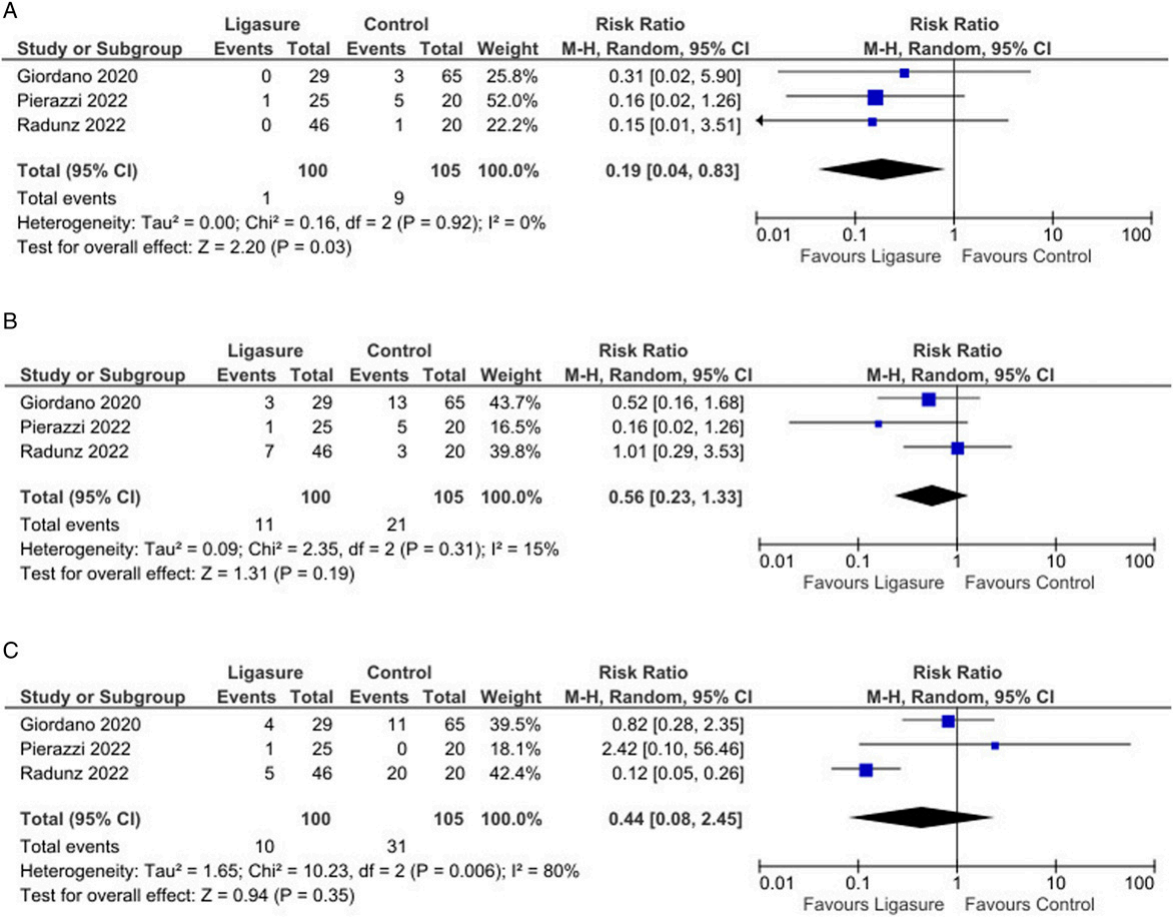
(A): Forest plot showing a significantly decreased occurrence of hematomas by using Ligasure compared with control; (B): Forest plot did not show any significant difference in the seroma occurrence by using Ligasure compared with control; (C): Forest plot did not show any significant difference in the infection occurrence by using Ligasure compared with control.

Likewise, these studies showed a significant reduction in postoperative hematoma incidence with LS (Risk Difference = −0.07, 95% CI: −0.15 to −0.01, *P* < 0.001, I^2^ = 36%; Risk Ratio = 0.19, 95% CI: 0.04 to 0.83, *P* = 0.03; Figure 2(A)). The absolute risk reduction for hematoma occurrence was 0.076, corresponding to an NNT of 14. This means that treating 14 patients with LS would prevent 1 hematoma compared to traditional methods.

**Figure 3.**
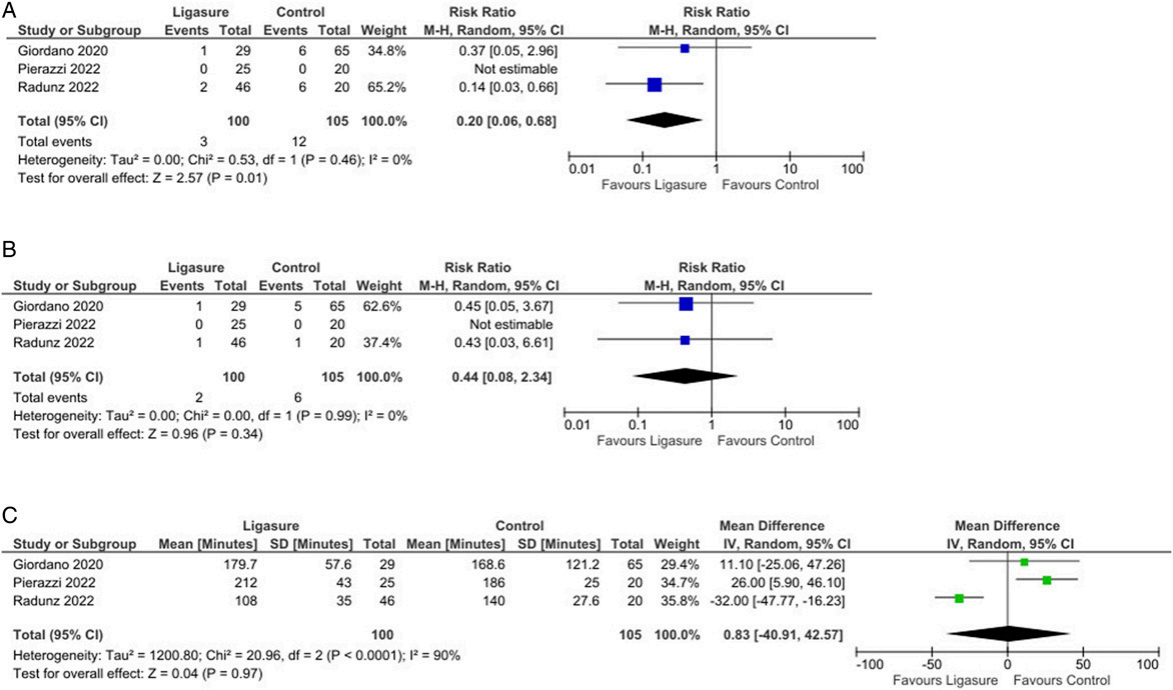
(A): Forest plot showing a significantly decreased occurrence of wound dehiscence by using Ligasure compared with control; (B): Forest plot did not show any significant difference in the fat or flap necrosis occurrence by using Ligasure compared with control; (C): Forest plot did not show any significant difference in the operative time by using Ligasure compared with control.

No significant differences were found between the groups regarding seroma incidence (*P* = 0.19, Figure 2(B)) or infection rates (*P* = 0.35, Figure 2(C)). However, analysis indicated a significantly lower incidence of wound dehiscence in the LS group compared to controls (Risk Ratio = 0.20, 95% CI: 0.06 to 0.68, *P* = 0.01; Figure 3(A)).

**Figure 4.**
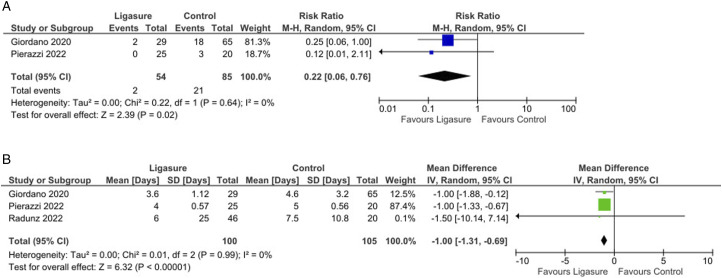
(A): Forest plot showing a significantly decreased rate of re-operation by using Ligasure compared with control; (B): Forest plot showing a significant difference in the length of hospital stay by using Ligasure compared with control.

The pooled analysis showed no significant reductions in fat or flap necrosis (*P* = 0.34, Figure 3(B)), nor were there differences in operative time (*P* = 0.97, Figure 3(C)). Interestingly, there was a significant reduction in re-operation rates (Risk Ratio = 0.22, 95% CI: 0.06 to 0.76, *P* = 0.02; Figure 4(A)) and a shorter hospital stay (Mean Difference = −1.00, 95% CI: −1.31 to −0.69, *P* < 0.001; Figure 4(B)) in favor of the LS group.

Significant statistical heterogeneity (I^2^ >75%) was noted in only 2 analyses (Figures 1(C) and 3(C)); in all other cases, heterogeneity was low (mostly I^2^ <25%). Sensitivity analyses confirmed that removing any individual study did not substantially alter the summary estimates. Quality details of the included studies are provided in Supplemental Table S1.

## Discussion

This study provides compelling current evidence on the benefits of LigaSure Impact™ in reducing specific postoperative complications in abdominoplasty after massive weight loss. Our findings indicate that LS was associated with significantly lower overall complication rates, particularly in the incidence of hematoma and wound dehiscence, compared to traditional dissection techniques. These results suggest that LS may offer substantial improvements in postoperative outcomes by enhancing hemostasis, reducing intraoperative bleeding, and promoting faster wound healing.

While outpatient abdominoplasty is now common practice for low-risk patients, this approach is often unsuitable for individuals who have undergone massive weight loss. In this population, the presence of comorbidities and the need for extensive tissue resection frequently necessitate inpatient monitoring. A notable finding was the significant reduction in re-operation rates and shorter hospital stays in patients where LS was used, highlighting its potential to reduce both health care costs and postoperative patient morbidity. These benefits are likely attributable to LS’s ability to provide consistent vessel sealing, minimizing blood loss and thereby reducing the need for postoperative interventions. This is particularly advantageous in the context of complex reconstructive surgeries following significant weight loss, where tissue integrity is often compromised, and postoperative complications are more common.

While LS demonstrated clear advantages in reducing hematomas and wound dehiscence, it did not significantly affect the incidence of seromas, infections, fat necrosis, or operative time compared to conventional techniques. These findings may reflect the multifactorial etiology of these complications, which may not be directly influenced by surgical hemostasis alone.

No significant differences were found in rates of flap or fat necrosis; however, wound dehiscence was notably higher with conventional flap dissection techniques in abdominoplasty (Figure 3(A) and (B)). The literature indicates that traditional electrosurgery can increase the risk of excessive thermal injury and necrosis, which may contribute to delayed wound healing and a higher incidence of seroma due to intraoperative thermal damage to lymphatic vessels.^34-36^ This inflammatory response, caused by tissue destruction and vessel coagulation, compromises blood supply, delays fibroblast migration, and impairs collagen formation, ultimately hindering wound healing injuries and related complications, advanced technologies for tissue dissection and coagulation have been introduced. In addition to alternatives like gaseous argon and ultrasound, the electrothermal bipolar vessel-sealing system (like LS) was developed for targeted control of vascular and lymphatic structures. This device applies radiofrequency energy precisely to tissue bundles and vascular structures, modulating the energy and pressure to minimize thermal spread and collateral tissue damage, thereby improving surgical outcomes.^37^

The application of LS in plastic surgery is relatively novel and has been described in only a limited number of reports. Previous studies have evaluated LS’s effectiveness for endoscopic latissimus dorsi harvesting^23^ and neurofibroma removal,^24^ both of which reported decreased bleeding, reduced operative time, and shorter hospital stays.

Beyond clinical outcomes, the economic implications of using LS warrant consideration. The device incurs a per-use cost of approximately €400-500, which may appear substantial in comparison to conventional dissection tools. However, potential cost offsets could arise from improved intraoperative efficiency, reduced blood loss, shorter operative time, and lower rates of postoperative complications such as seroma and hematoma. These factors may contribute to shorter hospital stays and decreased need for follow-up interventions, potentially resulting in overall cost savings. While this review suggests that LS may offer both clinical and economic benefits, formal cost-effectiveness analyses are lacking. A comprehensive health economic evaluation, incorporating direct device costs, complication-related expenses, and recovery timelines, is needed to more accurately determine the value proposition of energy-based devices in body contouring procedures such as abdominoplasty.

In our meta-analysis, only 1 study reported a reduction in operative time with LS,^27^ whereas the other 2 studies found longer durations.^26,28^ This discrepancy may be specific to abdominoplasty, a procedure that involves extensive tissue dissection. Operative time is also likely influenced by the surgeon’s familiarity with the device, and the variability observed may, in part, reflect a learning curve associated with LS use. The potential impact of this learning curve should be considered when interpreting differences in operative time outcomes.

In procedures with such large fields, LS’s efficiency may stem from reduced time spent on hemostasis and vessel ligation, thus speeding up these aspects of the operation.^38^ We believe that a key technical advantage of LS in abdominoplasty is its ability to effectively seal all blood and lymphatic vessels throughout the procedure, independent of intraoperative blood pressure fluctuations. This comprehensive vessel sealing significantly reduces the likelihood of unsealed vessels and subsequent postoperative bleeding.

The clinical application of LS in abdominoplasty should be considered in the context of individual patient factors and surgical goals. Patients who may derive the greatest benefit include those undergoing post-bariatric or massive weight loss body contouring, where extensive tissue dissection and vascularity increase the risk of blood loss and seroma formation. LS’s ability to seal vessels up to 7 mm and provide consistent hemostasis may be particularly advantageous in these cases.^39^ Surgeons considering adoption of this technology should weigh the potential benefits, such as reduced operative time, lower drain output, and fewer complications, against the cost and availability of the device. Training requirements are modest, as the device is intuitive and widely used in other surgical specialties; however, a short learning curve exists, and proficiency with energy-based dissection should be ensured before routine use in abdominoplasty. While no specific contraindications to LS use were reported in the reviewed studies, surgeons may prefer traditional methods in cases where tactile feedback or more delicate dissection is required, such as in revision procedures or patients with atypical anatomy. Ultimately, the choice of dissection tool should be guided by patient characteristics, surgeon experience, and especially resource availability, and further evidence from randomized studies will help refine these recommendations.

The findings of this meta-analysis should be interpreted with consideration of several limitations and potential biases that may influence the generalizability of the results. Only 3 studies, none of which were randomized controlled trials, were available and, therefore, used for this pooled analysis (Table 1). While none of these studies reported any industry sponsorship that might introduce bias, this lack of randomized data remains a limitation.

This systematic review is subject to several important limitations that warrant cautious interpretation of the findings. All included studies were retrospective and non-randomized in design, contributing to a high risk of bias across multiple domains. The total sample size was limited to 205 patients, and there was notable clinical heterogeneity in surgical techniques, perioperative protocols, and outcome measures. Additionally, none of the included studies adjusted for potential confounding variables, further limiting the strength of the conclusions that can be drawn.

Another key limitation of this review is the substantial clinical heterogeneity among the included studies, which impacts the interpretability and generalizability of our findings. The studies varied considerably in their control techniques (eg, scalpel vs electrocautery), surgical approaches, closure methods, and postoperative management protocols. Additionally, the patient populations differed in terms of baseline characteristics and comorbidities, which could influence outcomes such as wound healing, seroma formation, and complication rates. These variations introduce confounding factors that limit the ability to draw definitive conclusions or conduct meaningful subgroup analyses. Due to the small number of studies and incomplete reporting, stratified analysis was not feasible. Despite this heterogeneity, some outcomes, particularly reductions in intraoperative blood loss and postoperative drain output with the use of LS, were consistently observed across all studies, suggesting these findings may be more robust. Nonetheless, the methodological challenges of pooling data from diverse surgical techniques and patient populations underscore the need for caution in interpretation and highlight the importance of future high-quality randomized studies with standardized protocols.

Additionally, methodological heterogeneity in the extent of tissue undermining performed across the different studies could influence complication rates, adding further complexity to interpreting the outcomes.^8^ The use of drains and progressive tension sutures varied among included studies.

Unfortunately, all these factors were not consistently reported, limiting our ability to conduct subgroup analysis.

While the observed effect sizes in favor of LS appear substantial, they must be interpreted with caution. All included studies were positive 1 and they had small sample sizes, which increases the risk of random error and may contribute to overestimation of treatment effects.

A sensitivity analysis was performed by excluding the study identified as having the highest risk of bias to assess the robustness of the findings. However, given the small number of the studies included (n = 3) and their relatively small number of participants, this exclusion did not substantially alter the summary estimates.

All these factors underscore the preliminary nature and the fragility of the current evidence regarding the use of LS in abdominoplasty. To more definitively assess the safety and efficacy of this energy device in body contouring procedures, high-quality prospective studies, ideally randomized controlled trials, are needed to validate these initial findings and guide evidence-based surgical practice.

Key design recommendations include adequate randomization, blinding of outcome assessors, and clearly defined inclusion and exclusion criteria. Suggested primary endpoints may include intraoperative blood loss, operative time, and postoperative drain output, as these are direct measures of surgical efficiency and safety. Secondary endpoints should assess complication rates (eg, seroma, hematoma, infection), time to drain removal, patient satisfaction, and length of hospital stay. Sample size calculations should be based on expected differences in primary outcomes, with prior studies suggesting clinically meaningful reductions in blood loss and drain output; a power analysis should ensure detection of such differences with at least 80% power and a two-sided alpha of 0.05. To minimize bias and allow for comparability, future studies should implement standardized surgical protocols, particularly including flap elevation technique, dissection depth, closure method, and postoperative management (eg, drain usage, compression, pain catheters) and consider surgeon experience and learning curve.^25^ Multicenter trials would enhance generalizability, and incorporating a cost-effectiveness analysis would further increase clinical relevance.

Future research should also investigate additional outcomes, such as postoperative pain and patient satisfaction. The use of standardized aesthetic scoring systems and patient-reported outcome measures (PROMs) is essential to more comprehensively assess the clinical value of LS. Moreover, comparative studies involving other advanced energy devices, such as the harmonic scalpel, would be valuable in contextualizing LS’s performance within the broader landscape of surgical technologies.

## Conclusion

LS appears to be a valuable tool in abdominoplasty for massive weight loss patients, particularly in reducing complications that directly impact patient recovery and health care resources. Its consistent performance in minimizing hematomas and wound dehiscence suggests that LS could be beneficial as part of standard surgical practice in selecting patient populations. Further studies are needed to validate these initial findings and guide evidence-based surgical practice.

## Supplemental Material

Supplemental Material - Ligasure Impact™ to Reduce Complications After Abdominoplasty: A Meta-Analysis of Comparative Studies Supplemental Material for Ligasure Impact™ to Reduce Complications After Abdominoplasty: A Meta-Analysis of Comparative Studies by Salvatore Giordano, Veera Mäntylä, Andre’ Salval, Pietro G. di Summa, and Carlo Maria Oranges in Surgical Innovation
