# Supplementary material for: Ligasure Impact™ to Reduce Complications After Abdominoplasty: A Meta-Analysis of Comparative Studies
Source: Surg Innov. 2025 Aug 27;33(1):71–80. doi: 10.1177/15533506251374484 (PMC12738970; doi:10.1177/15533506251374484)
Supplement: Supplemental Material - Ligasure Impact™ to Reduce Complications After Abdominoplasty: A Meta-Analysis of Comparative Studies [file sj-pdf-1-sri-10.1177_15533506251374484.pdf]

Table S1. Supplemental Digital Content - Risk of Bias Assessments

Risk of Bias of RCTs Using Cochrane Collaboration's Risk of Bias Assessment tool. [30]

**First Author**	**Sequence generation**	**Allocation Concealment**	**Blinding**	**Incomplete outcome data**	**Selective outcome reporting**	**Other sources of bias**
Giordano 2020 [26]	Unclear	Unclear	High	High	High	Unclear
Radunz 2022 [27]	Unclear	Unclear	High	High	High	Unclear
Pierazzi 2022 [28]	Unclear	Unclear	High	High	High	Unclear

Risk of Bias of Non-RCTs Using Newcastle-Ottawa Scale. [31]

**First Author**	**Selection**	**Comparability**	**Outcome**	**Total**
Giordano 2020 [26]	**	****	***	9
Radunz 2022 [27]	**	****	***	9
Pierazzi 2022 [28]	**	****	***	9
